# Clinicopathological features associated with CD44 and CD63 expression in breast cancer

**DOI:** 10.3332/ecancer.2024.1779

**Published:** 2024-09-26

**Authors:** Carlos A. Castaneda, Miluska Castillo, Joselyn Sanchez, Luis Bernabe, Katherin Tello, Nancy Suarez, Raul Alatrista, Ximena Quiroz-Gil, Alexandra Granda-Oblitas, Javier Enciso, Nathaly Enciso, Henry L Gomez

**Affiliations:** 1 Faculty of Health Sciences, Universidad Cientifica del Sur, Lima 15067, Peru; 2 Department of Medical Oncology, Instituto Nacional de Enfermedades Neoplasicas, Lima 15038, Peru; 3 Department of Research, Instituto Nacional de Enfermedades Neoplasicas, Lima 15038, Peru; 4 Laboratorio de Células Madre, Universidad Cientifica del Sur, Lima 15067, Peru; 5 Direccion General de Investigacion, Desarrollo e Innovacion, Universidad Científica del Sur, Lima 15067, Peru; 6 Unidad de Ensayos Clinicos, Oncosalud-AUNA, Lima 15038, Peru

**Keywords:** breast cancer, CD44, CD63, tumor-infiltrating lymphocytes

## Abstract

**Background:**

CD44 is a cell-surface transmembrane glycoprotein that participates in the regulation of many cellular processes, including cell division, adhesion, migration and stem-like characteristics. CD63 is involved in the exocytosis process.

**Objective:**

To evaluate the relationship between CD44 and CD63 expression and clinicopathological features, including tumor-infiltrating lymphocytes (TILs), phosphoinositide 3-kinase (PIK3CA) mutation and survival.

**Methodology:**

CD44 and CD63 were stained in samples from 101 breast cancer cases from Peruvian women.

**Results:**

Median age was 52 years, most were most were grade-3 (68%), estrogen receptor (ER)+ (64%) and stage II-III (92%). Median ki67 was 30%, median stromal TIL was 30% and *PIK3CA* mutation was found in 49%. Longer survival was associated with earlier stages (*p* = 0.016), lower ki67 (*p* = 0.023), ER+ (*p* = 0.034), luminal phenotype (*p* = 0.029) and recurrence (*p* < 0.001). CD44 was classified as high cell density staining in 57% and high intensity in 55%. High CD44 density was associated with younger age (*p* = 0.043), triple-negative phenotype (*p* = 0.035) and shorter survival (*p* = 0.005). High CD44 expression was associated with short survival (*p* = 0.005). High CD63 cell density was found in 56% of cases and was associated with ER-positive (*p* = 0.045), low TIL levels (*p* = 0.007), Luminal-A (*p* = 0.015) and low CD44 intensity (*p* = 0.032).

**Conclusion:**

CD44 expression was associated with aggressive features and low CD63 density staining.

## Background

Breast cancer is the most common malignancy among women worldwide, including in Peru [1]. The clinicopathologic features that predict survival serve as prognostic factors. These include histologic features and the immunohistochemical status of estrogen-receptor (ER), progesterone-receptor (PgR) and HER2. Tumor-infiltrating lymphocytes (TIL) are a predictive biomarker for response to chemotherapy and immunotherapy, while phosphoinositide 3-kinase (*PIK3CA*) mutations predict response to targeted therapy [2].

CD44 is a cell surface transmembrane glycoprotein involved in regulating many cellular processes, including cell division, adhesion and migration by binding to its primary ligand, the hyaluronic acid [3, 4]. It promotes carcinogenesis by accelerating proliferation or suppressing apoptosis through the stimulation of Ras-Raf-Mek-Erk-cyclin D1 signaling and the *PIK3CA* pathway [4].

CD44 is also involved in the epithelial-mesenchymal-transition (EMT) process and in extravasation through the endothelial barrier, both of which stimulate tumor invasion and metastasis [3, 5–9]. Additionally, CD44-expressing breast cancer cells exhibit stem-cell-like characteristics and indicate a poor prognosis when detected within the bone marrow of patients with early-stage breast cancer [8–10]. Laboratory tests remarkably found that the knockdown of CD44 reduces tumor cell adhesion to endothelial cells *in vitro*, and decreases overall tumor burden in the brain, lung, liver and skeleton as well as increased cell survival *in vivo* experiments [11]. Despite this experimental data, there is no consensus on the association between CD44 expression and clinicopathological features [12–14]. Furthermore, there is no information on its expression in breast cancer series from South American countries.

Breast cancer patients have a higher number of extracellular vesicle proteins in plasma than healthy controls. The tetraspanin protein CD63 is a key factor in extracellular vesicle production and endocytosis [15]. It has been implicated in cell motility and metastatic capacity in cancer. Additionally, recent studies suggest that CD63 expression could also serve as a marker for stem cell features [16, 17]. Laboratory studies find that the exocytosis process could mediate immunosupression in the tumoral micro environment, and gene-modified mice with tetraspanin deficiency exhibit immune response alterations [18]. Unfortunatelly, few studies have evaluated the association between CD63 expression and clinical features or survival in breast cancer [15].

Additionally, different studies indicate that race acts as a prognostic factor. For instance, black women often have a worse prognosis, whereas Latina women may have a better prognosis. Various series also describe that breast tumors in Latinas have particular features, such as a younger age at diagnosis and a higher prevalence of triple-negative breast cancer (TNBC) phenotype. However, there is a lack of information about what molecular variants produce these differences [19, 20].

In this study, we evaluated the correlation between the expression of CD44, CD63 and clinicopathological features, including the level of TIL and *PIK3CA* mutations, as well as survival in a Peruvian breast cancer series.

## Materials and methods

### Patient selection

This is a retrospective study from a single institution. Patients with invasive breast cancer diagnosed between 2016 and 2018 and who have paraffin samples stored at the Instituto Nacional de Enfermedades Neoplasicas were included. The study was approved by the Ethics Committee. The clinicopathological information was obtained from the patients’ files. Additionally, an experienced pathologist reviewed the Hematoxylin Eosin (HE)-stained pathology slides to document missing histopathology data, including TILs according to the international TILs working group recommendation (JS) [21].

### Evaluation of CD44 and CD68 staining in paraffin samples

Tissue microarrays (TMAs) were constructed from tumor areas obtained from paraffin blocks with 6.0 mm diameter cores. Serial 4 μm sections were prepared and used for immunohistochemical (IHC) staining. Sections of paraffin samples were rehydrated in phosphate-buffered saline (PBS) and immersed in 0.1% trypsin solution in PBS at 37°C for 5 to 10 minutes or microwaved for 5 minutes for 4 (total, 20 minutes) in buffer to recover the antigen. Sections were treated for 45 minutes with 10% normal goat serum or normal horse serum in PBS. The primary anti-human antibodies used for IHC (following manufacturer’s instructions) were rabbit anti-CD44 monoclonal antibody (clone SP37, MAD-000537QD, ready-to-use, Vitro Master Diagnostica, Granada, Spain) and CD63 (clone NKI/C3, MAD-000543QD, ready-to-use, Vitro Master Diagnostica, Granada, España). Sections were incubated in alkaline phosphatase-streptavidin (Vector Laboratories, Burlingame, CA; 1:1,000 dilution) for 30 minutes at room temperature, and reacted with Fast-Red Substrate System (Dakopatts) and Dako^®^ Fuchsin + Substrate- Chromogen. Background staining was performed with Mayer’s hematoxylin solution, and then sections were dehydrated through rising alcohols to xylene and mounted on slides.

**Figure 1.**
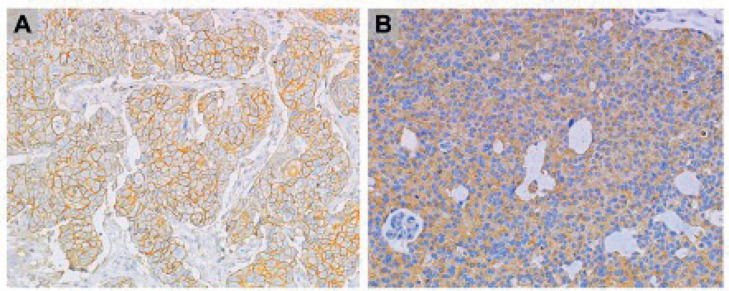
(A): CD44 and (B): CD63 IHC staining at 20× magnification.

The stained slides were read by a pathologist (JS) and categorized into three categories, status, density and intensity. Density was classified as high when brown staining was found in at least 10% (median value and previously described cutoff [22, 23]) of cancer cells with the anti-CD44 reactive, and when at least 80% (median value) of cancer cells were stained with anti-CD63 slides. The intensity of CD44 and CD63 was high when the darker degree of staining was observed in the nucleus and membrane locations, respectively (Figure 1).

### Detection of PIK3CA mutation

DNA was extracted from paraffin-embedded tumor samples using the QIAamp DNA FFPE tissue kit (Qiagen; Hilden, Germany) [24]. TaqMan-based real-time PCR analysis was carried out using the LightCycler^®^ 96 PCR System (Roche Applied Science, Mannheim, Germany) to detect the three ‘hot spot’ *PIK3CA* mutations (H1047R, E545K and E542K). Custom TaqMan primers and probes were designed for *PIK3CA* mutations (*PI3KCA* 760: c.1624 G (VIC) >A (FAM), *PI3KCA* 763: c.1633 G (VIC) >A (FAM), *PI3KCA* 775: c.3140 A (VIC) >G (FAM), Scientific ThermoFisher). The thermal cycler protocol was: 10 minutes at 96°C, 39 cycles at 60°C for 2 minutes, 98°C for 30 seconds and 60°C for 1 minutes. Each mutation was analysed in a single assay for all samples. The mutational threshold was determined to be 1% after measuring the WT HDx FFPE reference standards (Horizon Diagnostics, Cambridge, United Kingdom). On the other hand, the threshold for reagents that induced false positives was assumed to be 0.5% of the mutation frequency.

### Statistical analysis

Relationships between expression status and clinicopathological features were detected using Chi-square or Fisher’s exact tests. Median follow-up was 5.48 years, and survival rates were calculated from diagnosis to recurrence or last follow-up date/death, and estimated using the Kaplan-Meier method. *p* values less than 0.05 were considered statistically significant. Statistical analyses were performed using the Statistical Package for the Social Sciences (SPSS) for Windows version 26.0 (SPSS Inc., Chicago, IL, USA).

## Results

### Clinical and pathological features

It included 101 cases of breast cancer in Peruvian women and the median age was 52 years. Most had NST histology (94%), grade 3 (68%), ER-positive (64.4%), luminal-B phenotype (45.5%), TNBC (21.8%) and stage II–III (92%). Carcinoma *in-situ* was found in 65 cases (65%). The median ki67 was 30%, the median stromal TIL was 30%, and the *PIK3CA* mutation was found in 49% (*n* = 49). Only one mutation was found in 27 cases (14 cases with H1047R, 8 with E545K and 5 with E542K). Two mutations were found in 15 (9 cases with H1047R and E545K, 1 with H1047R and E542K and 5 with E542K and E545K). The three mutations were found in seven cases.

**Figure 2.**
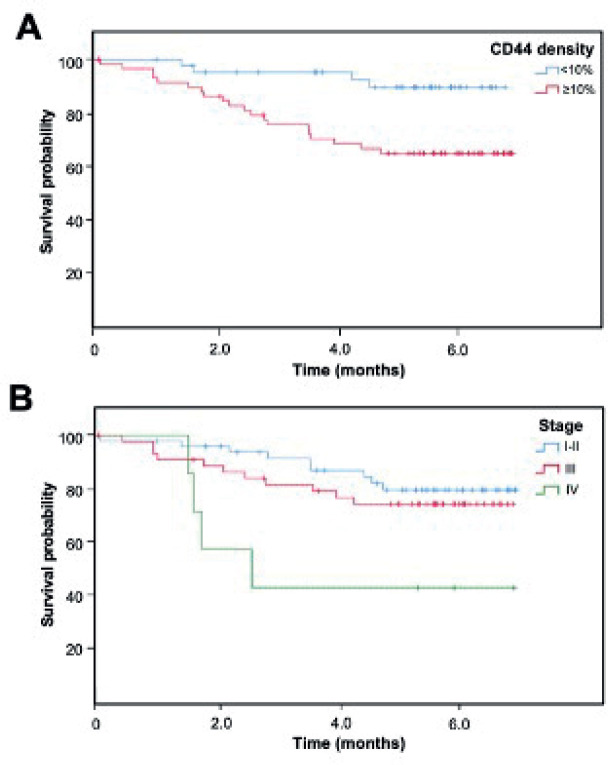
Kaplan-Meier overall survival curve according to (A) CD44 density and (B) clinical stage.

Recurrence was found in 21 cases and death in 26. Longer overall survival was associated with earlier stages (*p* = 0.016), lower ki67 (*p* = 0.023), ER+ (*p* = 0.034), luminal phenotype (*p* = 0.029) and the absence of recurrence (p < 0.001) (Table 1, Figure 2).

### Relationship between CD44 and tumor features

CD44 was classified as positive status in 77 (76.2%), high density (larger or equal than 10%) in 58 (57.4%) and high intensity in 56 (55.4%) (Table 1). The high density of CD44 was associated with younger age (*p* = 0.043), triple-negative (*p* = 0.035), shorter overall survival (*p* = 0.005) and a trend for RE-negative status (*p* = 0.162). CD44 status was not associated with the presence of *in-situ* carcinoma, TIL levels, *PIK3CA* mutations (*p* = 0.898) nor any of the three specific mutations (*p* = 0.144–0.862) (Table 2, Figure 2).

### Relationship between CD63 and tumor features

CD63 was classified as positive status in 77 (81.9%), high density (larger or equal to the median value, 80%) in 44 (56.4%) and high intensity in 27 (30%). High density was associated with the absence of both lymphovascular invasion (*p* = 0.045) and perineural invasion (*p* = 0.044), as well as with ER-positive (*p* = 0.045) and low TIL levels (*p* = 0.007). High CD63 intensity was associated with Luminal-A (52.6% *vs.* 23.9%, *p* = 0.015) and with non-Luminal-B (20% *vs.* 40%, *p* = 0.038) phenotypes.

High CD63 density was associated with low CD44 intensity (*p* = 0.032).

CD63 expression was not associated with the presence of *in-situ* carcinoma, *PIK3CA* mutations (*p* = 0.789) nor any of the three mutations (*p* = 0.308–0.887) (Table 3).

## Discussion

Our analysis showed that high CD44 protein expression was associated with young age, TNBC phenotype and shorter survival. As previously mentioned, laboratory experiments indicate that CD44 is a CSC marker, an important up-regulator of the EMT process, and promotes metastasis and chemotherapy resistance [3, 25]. Other tumor sample analysis have also described this aggressive feature of CD44 staining. Gudadze et al. found that CD44 protein was associated with ER-negative status, luminal-B and TNBC phenotypes as well as larger tumor size and node involvement in their series of 393 breast cancer series [26]. Similarly, McFarlane et al., described that CD44 expression was associated with high grade (*p* = 0.046), ER-negative (*p* = 0.001), PgR-negative status (*p* = 0.029) and higher recurrence rates in their 448 breast cancer cases [11]. Similarly, other groups have also found an association between CD44 and the TNBC phenotype [27, 28].

Our finding of an association between CD44-positive and shorter survival has also been described by other groups. McFarlane et al. found that CD44 expression was correlated with increased distant recurrence and reduced disease-free survival in patients with lymph node-positive or large tumors [11]. Finally, a systematic review including nine studies found that CD44-positive was associated with inferior survival, larger tumor size, higher grade and node involvement in TNBC [29].

Our analysis did not find that CD44 expression modulates TIL levels, despite the fact that CD44 expression has recently been linked with the increased expression of PD-L1 in TNBC cells [30]. We also did not find that co-presence of *in-situ* cancer cells would be associated with overexpression of CD44, as previously published [31].

Finally, previous studies have described that populations with extensive African ancestry have high rates of tumors with stem cell features as well as TNBC rates [32, 33]. Therefore, our findings could also suggest that the high prevalence of TNBC in our Latinas series could be associated with high rates of stem cell tumor features in this population [20, 34].

On the other side, our finding of an association between the expression level of the vesicle protein CD63 and all favorable prognostic features—ER-positive, Luminal-A phenotype, low TIL levels and low CD44 (a marker of stem-cell features)—has not been previously described in breast cancer.

Other series have also described that CD63 levels are inversely related to cancer invasion and metastases [35]. Similarly, Huang *et al* [36] describe that CD63 expression inhibits proliferation, migration, EMT and stemness in head and neck cancer. The relationship between tetraspanins and stem cell features has been previously described, as vesicles are vehicles for transmitting self-renewing and multilineage differentiating capabilities among cells [37]. Our finding that Luminal-A and ER-positive tumors have higher CD63 expression could indicate that vesicles could also transmit information from this group of cancer cells.

Our results differ from other publications that indicate CD63 levels and other tetraspanins are associated with aggressive features and poor prognosis in various malignancies [38–40] including breast cancer [41, 42]. This opposite finding could be related to the evaluation technique.

Our results found some associations between features of breast cancer and biomarkers related to stem cell features and cell vesicle production. This information has been generated in women from South America, and recent studies in precision medicine indicate that genomic variants could predispose to particular tumor features in the Peruvian population [19, 20].

More South American populations need to be included in research evaluating biomarkers to increase the knowledge of this pathology.

### Conclusion

High levels of CD44 are associated with shorter survival and TNBC phenotype. CD63 distribution is inversely associated with CD44 distribution. High CD63 cell density is also associated with ER-positive, low TIL levels and Luminal-A phenotype.

## Conflicts of interest

The authors declare that they have no conflict of interest.

## Institutional review

The conduct of this survey was approved by The Institutional Review Board of INEN (#INEN-16-30). Since the study was based on a secondary source and there was no contact with the patients, no informed consent was applied; however, the identity and personal data of patients’ medical records were protected at all times.

## Figures and Tables

**Table 1.** Clinicopathological features and outcomes.

Variable	Total *N* (%)	N° de eventos	OS-5y	*p*
Total	101 (100%)	24	76.2	
Age, year				0.666
< 52	47 (46.5)	12	74.5	
≥ 52	54 (53.5)	12	77.9	
Stage				0.016
I	1 (1.0)	1	0.0	
II	48 (47.5)	8	83.3	
III	45 (44.6)	11	75.6	
IV	7 (6.9)	4	42.9	
Histology				0.438
Ductal/NST	95 (94.0)	22	76.8	
Other	6 (5.9)	2	66.7	
Grade				0.067
1-2	32 (31.7)	4	87.5	
3	69 (68.3)	20	71.0	
ILV				0.439
No	41 (40.6)	9	78.0	
Yes	55 (55.5)	15	72.7	
IPN				0.323
No	68 (70.8)	15	77.9	
Yes	28 (70.8)	9	67.9	
Ki67				0.023
<30%	43 (43.0)	6	86.0	
≥30%	57 (57.0)	18	68.4	
Phenotype				0.029
Luminal-A	21 (20.8)	19	90.5	
Luminal-B	46 (45.5)	35	76.1	
HER-2	12 (11.9)	10	83.3	
TNBC	22 (21.8)	13	59.1	
ER				0.034
Negative	36 (35.6)	12	66.7	
Positive	65 (64.4)	12	81.5	
HER2				0.619
Negative	72 (71.3)	18	75.0	
Positive	26 (28.7)	6	76.9	
PgR				0.057
Negative	44 (43.6)	14	68.2	
Positive	57 (56.4)	10	82.5	
*PIK3CA* mutation				0.083
Negative	51 (51.0)	9	82.4	
Positive	49 (49.0)	15	69.4	
TILs				0.409
<30%	43 (43.4)	8	81.4	
≥30%	56 (56.6)	14	75.0	
CD44 status				0.059
Negative	24 (23.8)	2	91.7	
Positive	77 (76.2)	22	71.4	
CD44 density				0.005
<10%	43 (42.6)	4	90.7	
≥10%	58 (57.4)	20	65.5	
CD44 intensity				0.143
Low	45 (44.5)	8	82.2	
High	56 (55.4)	16	71.4	
CD63 status				0.576
Negative	17 (18.1)	3	82.4	
Positive	77 (81.9)	19	75.3	
CD63 density				0.866
<80%	34 (43.6)	8	76.5	
≥80%	44 (56.4)	11	75.0	
CD63 intensity				0.386
Low	63 (70.0)	16	74.6	
High	27 (30.0)	5	81.5	
Recurrence				<0.001
No	80 (82.2)	9	85.5	
Yes	21 (17.8)	15	27.8	

**Table 2.** Relationship between CD44 and clinicopathological features.

Features	CD44 status			CD44 density			CD44 Intensity		
	Negative *n* (%)	Positive *n* (%)	*p*	<10% *n* (%)	≥10% *n* (%)	*p*	Low *n* (%)	High *n* (%)	*p*
Total	24 (23.8)	77 (76.2)		43 (42.6)	58 (57.4)		45 (44.5)	56 (55.4)	
Age, year			0.051			0.043			0.047
<52	7 (14.9 )	40 (85.1)		15 (31.9)	32 (68.1)		16 (34.0)	31 (66.0)	
≥52	17 (31.5)	37 (68.5)		28 (51.9)	26 (48.1)		29 (53.7)	25 (46.3)	
Stage			0.941			0.68			0.737
I	0 (0.0 )	1 (100.0)		0 (0.00)	1 (100.0)		0 (0)	1 (100)	
II	12 (25.0)	36 (75.0)		20 (41.7)	28 (58.3)		21 (43.8)	27 (56.3)	
III	10 (22.2 )	35 (77.8)		20 (44.4)	25 (55.6)		20 (44.4)	25 (55.6)	
IV	2 (28.6 )	5 (71.4)		3 (42.9)	4 (57.1)		4 (57.1)	3 (42.9)	
Histology			0.676			0.931			0.962
Ductal/NST	22 (23.2)	73 (76.8)		40 (42.1)	55 (57.9)		42 (44.2)	53 (55.8)	
Lobular	1 (25.0)	3 (75.0)		2 (50.0)	2 (50.0)		2 (50.0)	2 (50.0)	
Otro	1 (50.0)	1 (50.0)		1 50.0	1 (50.0)		1 (50.0)	1 (50.0)	
Grade			0.599			0.245			0.281
1	1 (50.0 )	1 (50.0)		2 (100.0)	0 (0.0)		2 (100)	0 (0)	
2	6 (20.0 )	24 (80.0)		13 (43.3)	17 (56.7)		13 (43.3)	17 (56.7)	
3	17 (24.6 )	52 (75.4)		28 (40.6)	41 (59.4)		30 (43.5)	39 (56.5)	
ILV			0.493			0.534			0.274
No	8 (19.5)	33 (80.5)		19 (46.3)	22 (53.7)		21 (51.2)	20 (48.8)	
Yes	14 (25.5)	41 (74.5)		22 (40.0)	33 (60.0)		22 (40.0)	33 (60.0)	
IPN			0.824			0.179			0.807
No	16 (23.5)	52 (76.5)		32 (47.1)	36 (52.9)		31 (45.6)	37 (54.4)	
Yes	6 (21.4)	22 (78.6)		9 (32.1)	19 (67.9)		12 (42.9)	16 (57.1)	
ER			0.448			0.162			0.091
Negative	7 (19.4)	29 (80.6)		12 (33.3)	24 (66.7)		12 (33.3)	24 (66.7)	
Positive	17 (26.2)	48 (73.0)		31 (47.7)	34 (52.3)		33 (50.8)	32 (49.2)	
PgR			0.247			0.146			0.146
Negative	8 (18.2)	36 (81.8)		16 (36.4)	28 (63.6)		16 (36.4)	28 (63.6)	
Positive	16 (28.1)	41 (71.9)		27 (47.4)	30 (52.6)		29 (50.9)	28 (49.1)	
HER2			0.628			0.325			0.463
Negative	16 (22.2)	56 (77.8)		28 (38.9)	44 (61.1)		30 (41.7)	42 (58.3)	
Positive	7 (26.9)	19 (73.1)		13 (50.0)	13 (50.0)		13 (50.0)	13 (50.0)	
Ki67			0.88			0.835			0.66
<30%	10 (23.3)	33 (76.7)		19 (44.2)	24 (55.8)		20 (46.5)	23 (53.5)	
≥30%	14 (24.6)	43 (75.4)		24 (42.1)	33 (57.9)		24 (42.1)	33 (57.9)	
Phenotype			0.622			0.186			0.211
Luminal-A	5 (23.8)	16 (76.2)		11 (52.4)	10 (47.6)		9 (42.9)	12 (57.1)	
Luminal-B	13 (28.3)	33 (71.7)		21 (45.7)	25 (54.3)		25 (54.3)	21 (45.7)	
HER2	3 (25.0)	9 (75.0)		6 (50.0)	6 (50.0)		5 (41.7)	7 (58.3)	
TNBC	3 (13.6)	19 (86.4)		5 (22.7)	17 (77.3)		6 (27.3)	16 (72.7)	
Triple-negative			0.207			0.035			0.065
No	21 (26.6)	58 (73.4)		38 (48.1)	41 (51.9)		39 (49.4)	40 (50.6)	
Yes	3 (13.6)	19 (86.4)		5 (22.7)	17 (77.3)		6 (27.3)	16 (72.7)	
*PIK3CA* Mutation			0.898			0.565			0.821
Negative	12 (23.5)	39 (76.5)		20 (39.2)	31 (60.8)		23 (45.1)	28 (54.9)	
Positive	11 (22.4)	38 (77.6)		22 (44.9)	27 (55.1)		21 (42.9)	28 (57.1)	
TILs			0.500			0.588			0.717
<30%	9 (20.9)	34 (79.1)		20 (46.5)	23 (53.5)		20 (46.5)	23 (53.5)	
≥30%	15 (26.8)	41 (73.2)		23 (41.1)	33 (58.9)		24 (42.9)	32 (57.1)	
Recurrence			0.568			0.336			0.86
No	20 (25.0)	60 (75.0)		36 (45.0)	44 (55.0)		36 (45.0)	44 (55.0)	
Yes	4 (19.0)	17 (81.0)		7 (33.3)	14 (66.7)		9 (42.9)	12 (57.1)	

**Table 3.** Relationship between CD63 and clinicopathological features.

Features	CD63 status			CD63 density			CD63 intensity		
	Negative *n* (%)	Positive *n* (%)	*p*	<80% *n* (%)	80% *n* (%)	*p*	Low *n* (%)	High *n* (%)	*p*
Total	17	77		34	44		63	27	
Age, year			0.904			0.733			0.097
<52	8 (18.6)	35 (81.4)		16 (45.7)	19 (54.3)		33 (78.6)	9 (21.4)	
≥52	9 (17.6)	42 (82.4)		18 (41.9)	25 (58.1)		30 (62.5)	18 (37.5)	
Stage			0.037			0.742			0.412
I	3 (60.0)	2 (40.0)		1 (50.0)	1 (50.0)		2 (66.7)	1 (33.3)	
II	11 (18.6)	48 (81.4)		19 (38.8)	30 (61.2)		36 (64.3)	20 (35.7)	
III	2 (7.4)	25 (92.6)		13 (52.0)	12 (48.0)		23 (82.1)	5 (17.9)	
IV	1 (33.3)	2 (66.7)		1 (50.0)	1 (50.0)		2 (66.7)	1 (33.3)	
Histology			0.493			0.735			0.091
Ductal/NST	17 (19.3)	71 (80.7)		32 (44.4)	40 (55.6)		60 (71.4)	24 (28.6)	
Lobular	0 (0.0)	4 (100.0)		1 (25.0)	3 (75.0)		1 (25.0)	3 (75.0)	
Otro	0 (0.0)	2 (100.0)		1 (50.0)	1 (50.0)		2 (100.0)	0 (0.0)	
Grade			0.798			0.131			0.72
1	0 (0.0)	2 (100.0)		1 (50.0)	1 (50.0)		1 (50.0)	1 (50.0)	
2	5 (18.5)	22 (81.5)		6 (26.1)	17 (73.9)		18 (66.7)	9 (33.3)	
3	12 (18.5)	53 (81.5)		27 (50.9)	26 (49.1)		44 (72.1)	17 (27.9)	
ILV			0.822			0.045			0.314
No	6 (16.7)	30 (83.3)		9 (29.0)	22 (71.0)		23 (63.9)	13 (36.1)	
Yes	10 (18.5)	44 (81.5)		23 (52.3)	21 (47.7)		37 (74.0)	13 (26.0)	
IPN			0.148			0.044			0.456
No	9 (14.1)	55 (85.9)		28 (49.1)	29 (50.9)		44 (72.1)	17 (27.9)	
Yes	7 (26.9)	19 (73.1)		4 (22.2)	14 (77.8)		16 (64.4)	9 (36.0)	
ER			0.822			0.045			0.842
Negative	6 (19.4)	25 (80.6)		15 (60.0)	10 (40.0)		20 (71.4)	8 (28.6)	
Positive	11 (17.5)	52 (82.5)		19 (35.8)	34 (64.2)		43 (69.4)	19 (30.6)	
PgR			0.977			0.095			0.573
Negative	7 (17.9)	32 (82.1)		18 (54.5)	15 (45.5)		24 (66.7)	12 (33.3)	
Positive	10 (18.2)	45 (81.8)		16 (35.6)	29 (64.4)		39 (72.2)	15 (27.8)	
HER2			0.384			0.405			0.816
Negative	10 (15.4)	55 (84.6)		22 (39.3)	34 (60.7)		43 (68.3)	20 (31.7)	
Positive	6 (23.1)	20 (76.9)		10 (50.0)	10 (50.0)		17 (70.8)	7 (29.2)	
ki67			0.669			0.079			0.184
<30%	8 (19.0)	34 (81.0)		11 (32.4)	23 (67.6)		25 (62.5)	15 (37.5)	
≥30%	8 (15.7)	43 (84.3)		23 (52.3)	21 (47.7)		37 (75.5)	12 (24.5)	
Phenotype			0.701			0.213			0.022
Luminal-A	2 (10.0)	18 (90.0)		5 (27.8)	13 (72.2)		9 (47.4)	10 (52.6)	
Luminal-B	10 (22.2)	35 (77.8)		15 (41.7)	21 (58.3)		26 (80.0)	9 (20.0)	
HER2	2 (16.7)	10 (83.3)		5 (50.0)	5 (50.0)		5 (50.0)	5 (50.0)	
TNBC	3 (17.6)	14 (82.4)		9 (64.3)	5 (35.7)		13 (81.3)	3 (18.8)	
TILs			0.742			0.007			0.211
<30%	8 (20.0)	32 (80.0)		9 (27.3)	24 (72.7)		26 (65.0)	14 (35.0)	
≥30%	9 (17.3)	43 (82.7)		25 (58.1)	18 (41.9)		37 (77.1)	11 (22.9)	
*PIK3CA* Mutation			0.789			0.842			0.713
Negative	9 (19.1)	38 (80.9)		17 (44.7)	21 (55.3)		30 (68.2)	14 (31.8)	
Positive	8 (17.0)	39 (83.0)		17 (42.5)	23 (57.5)		33 (71.7)	13 (28.3)	
CD44 status			0.989			0.495			0.83
Negative	4 (18.2)	18 (81.8)		7 (36.8)	12 (63.2)		15 (68.2)	7 (31.8)	
Positive	13 (18.1)	59 (81.9)		27 (45.8)	32 (54.2)		48 (70.6)	20 (29.4)	
CD44 intensity			0.748			0.032			0.519
Low	7 (16.7)	35 (83.3)		11 (30.6)	25 (69.4)		28 (66.7)	14 (33.3)	
High	10 (19.2)	42 (80.8)		23 (54.8)	19 (45.2)		35 (72.9)	13 (27.1)	
CD44 density			0.226			0.218			0.355
<10%	5 (12.5)	35 (87.5)		13 (36.1)	23 (63.9)		26 (65.0)	14 (35.0)	
≥10%	12 (22.2)	42 (77.8)		21 (50.0)	21 (50.0)		37 (74.0)	13 (26.0	
Recurrence			0.087			0.934			0.866
No	16 (21.6)	58 (78.4)		26 (43.3)	34 (56.7)		50 (70.4)	21 (29.6)	
Yes	1 (5.0)	19 (95.0)		8 (44.4)	10 (55.6)		13 (68.4)	6 (31.6)	
